# Site-specific glycoproteomic characterization of ES-62: The major secreted product of the parasitic worm *Acanthocheilonema viteae*

**DOI:** 10.1093/glycob/cwz035

**Published:** 2019-05-16

**Authors:** Simon J North, Kwamina Botchway, James Doonan, Felicity E Lumb, Anne Dell, William Harnett, Stuart M Haslam

**Affiliations:** 2 Department of Life Sciences, Imperial College London, London SW7 2AZ, United Kingdom; 3 Strathclyde Institute of Pharmacy and Biomedical Sciences, University of Strathclyde, Glasgow G4 0RE, United Kingdom

**Keywords:** ES-62, glycoproteomics, mass spectrometry, phosphorylcholine

## Abstract

ES-62 is the major secreted product of the parasitic filarial nematode *Acanthocheilonema viteae* and has potent anti-inflammatory activities as a consequence of posttranslational decoration by phosphorylcholine (PC). Previously, we showed that ES-62’s PC was attached to N-linked glycans, and using fast atom bombardment mass spectrometry, we characterized the structure of the glycans. However, it was unknown at this time which of ES-62’s four potential N-glycosylation sites carries the PC-modified glycans. In the present study, we now employ more advanced analytical tools—nano-flow liquid chromatography with high-definition electrospray mass spectrometry—to show that PC-modified glycans are found at all four potential N-glycosylation sites. Also, our earlier studies showed that up to two PC groups were detected per glycan, and we are now able to characterize N-glycans with up to five PC groups. The number per glycan varies in three of the four glycosylation sites, and in addition, for the first time, we have detected PC on the N-glycan chitobiose core in addition to terminal GlcNAc. Nevertheless, the majority of PC is detected on terminal GlcNAc, enabling it to interact with the cells and molecules of the immune system. Such expression may explain the potent immunomodulatory effects of a molecule that is considered to have significant therapeutic potential in the treatment of certain human allergic and autoimmune conditions.

## Introduction

ES-62 is the major secreted product of the parasitic rodent filarial nematode *Acanthocheilonema viteae* (Harnett et al. 1989). Parasitic worms as a group are known to secrete highly potent immunomodulatory molecules (reviewed by Harnett 2014), and consistent with this, comprehensive functional analysis of ES-62 has revealed it to modulate or impair the activities of a number of cells of the immune system (reviewed by Pineda et al. 2014). ES-62 achieves such effects by direct interaction with cells via specific receptors like Toll-like receptor 4 and subsequent subversion of associated cell signaling pathways (Pineda et al. 2014). The net effect of ES-62 is thus conversion of a pro-inflammatory to an anti-inflammatory immunological phenotype. As a consequence of this, ES-62 has been tested in a range of mouse models of allergic and autoimmune conditions and was found to offer protection against the development of lung and skin hypersensitivity (Melendez et al. 2007), arthritis (McInnes et al. 2003), systemic lupus erythematosus (SLE) (Rodgers et al. 2015) and the accelerated atherosclerosis associated with SLE (Aprahamian et al. 2015). For this reason, ES-62 is considered to have significant therapeutic potential against such conditions and toward this, novel synthetic drug-like small molecule analogues (SMAs), which mirror the parent molecules capabilities, have been successfully produced (Al-Riyami et al. 2013).

**Fig. 1:**
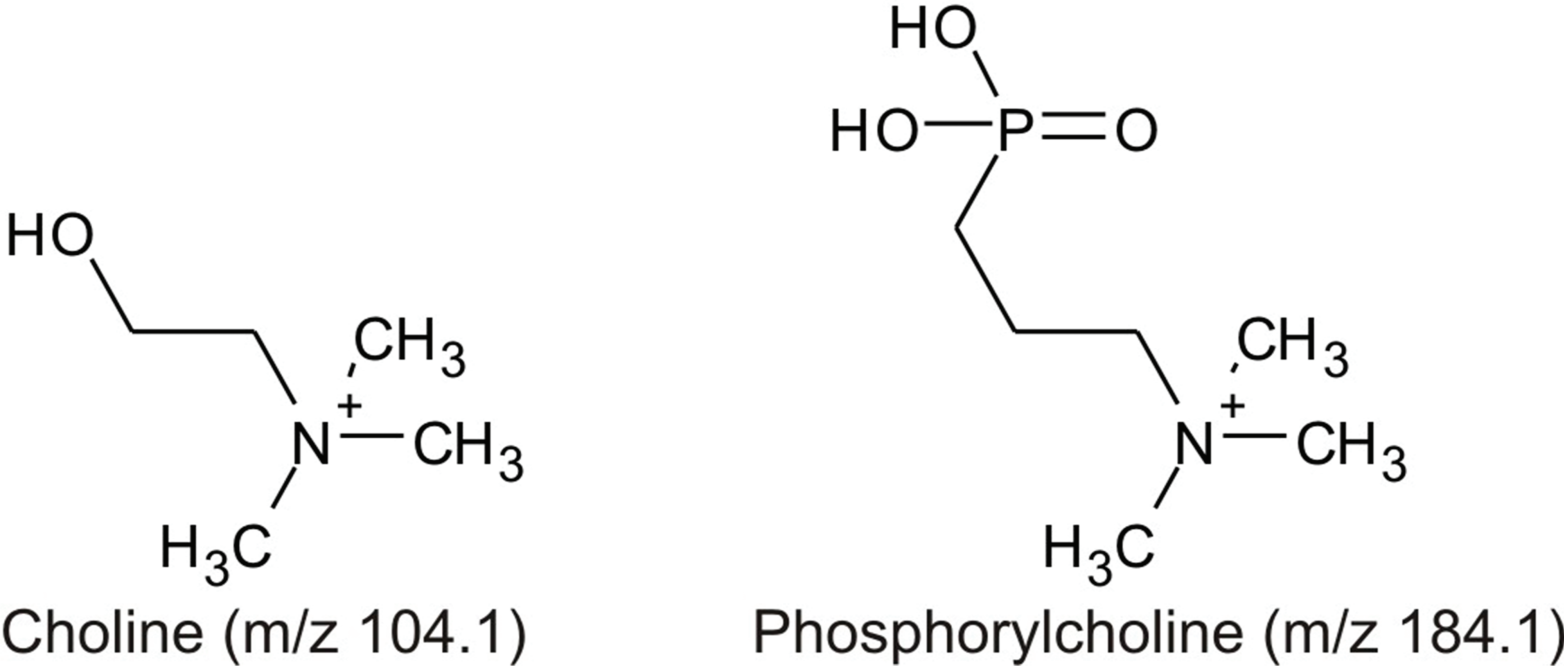
Characteristic fragment ion structures of choline (*m/z* 104.1) and PC (*m/z* 184.1) that were utilized to assist PC-containing glycopeptide identification (Timm et al. 2015). This figure is available in black and white in print and in colour at Glycobiology online.

**Fig. 2:**
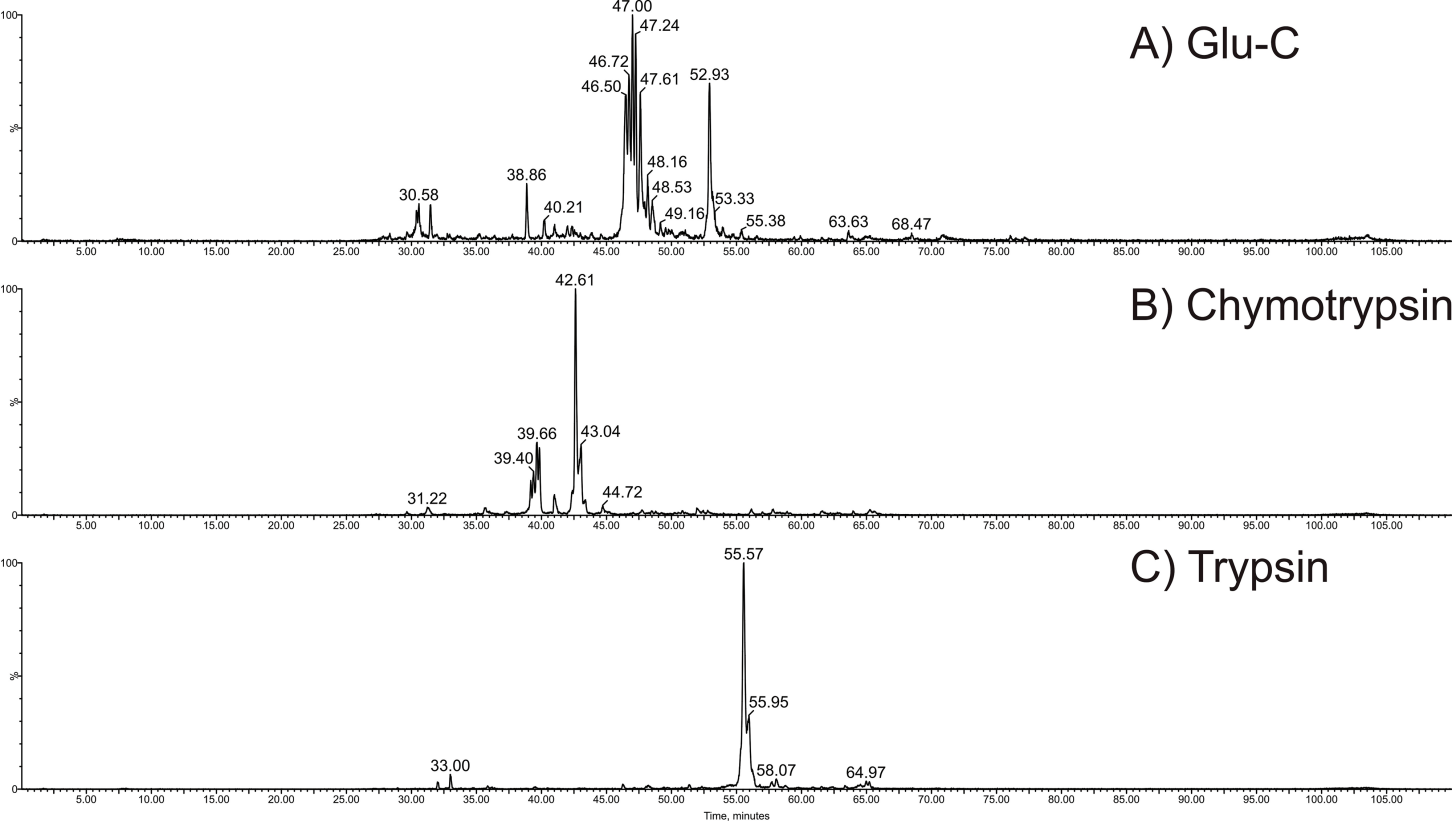
XICs searching for evidence of choline (*m/z* 104.1) and PC (*m/z* 184.1) marker ions in the MS/MS (MS^e^) analyses of ES-62 digests. (**A**) Reduced, carbamidomethylated Glu-C digest; (**B**) reduced, carbamidomethylated chymotrypsin digest; (**C**) reduced, carbamidomethylated trypsin digest. This figure is available in black and white in print and in colour at Glycobiology online.

**Fig. 3:**
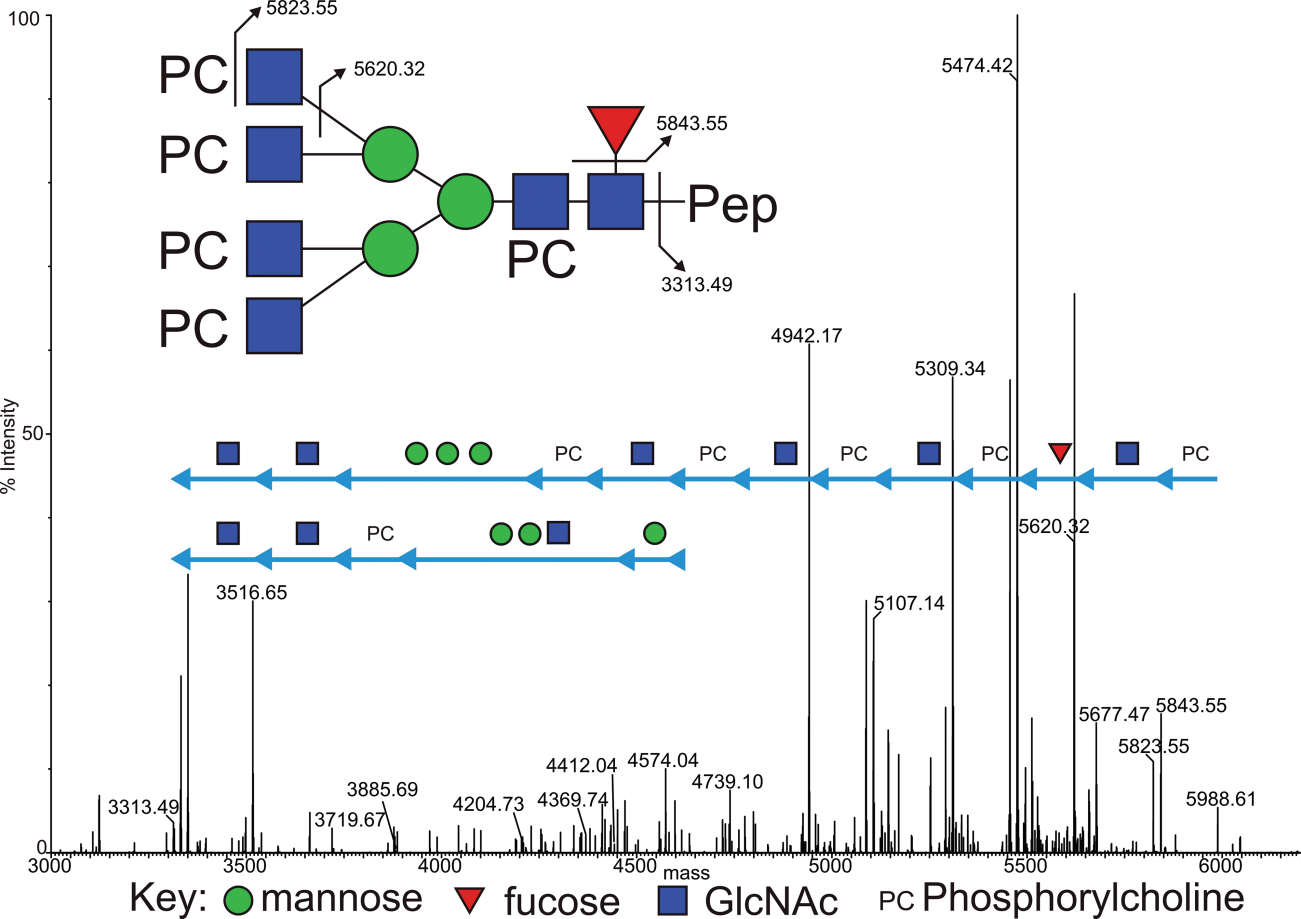
Deconvoluted MS/MS (MS^e^) spectrum of the m/z 5989.53 Asn 213 glycopeptide molecular ion, observed at a retention time of 55.8 min in the reduced, carbamidomethylated trypsin digest of ES-62. Focus is on the high-mass region, showing the sequential loss of glycan residues from the intact glycopeptide. This figure is available in black and white in print and in colour at Glycobiology online.

The amino acid sequence of ES-62 is consistent with it being an exopeptidase and indeed weak peptidase activity has been obtained when employing artificial substrates (Harnett et al. 1999). However, ES-62’s immunomodulatory properties are not due to such activity but rather to a posttranslational modification, the addition of PC (reviewed by Pineda et al. 2014). By the use of the enzyme *N*-glycosidase F and the inhibitor of initiation of *N*-type glycosylation, tunicamycin, it was possible to show that ES-62’s PC was attached via an *N*-linked glycans (reviewed by Harnett et al. 2010). Further work employing inhibitors of intracellular trafficking and oligosaccharide processing suggested that the substrate for PC addition was the 3-linked branch of Man_5_GlcNAc_3_ or Man_3_GlcNAc_3_ (Harnett et al. 2010). Consistent with this, subsequent fast atom bombardment mass spectrometry analysis of ES-62’s *N*-linked glycans suggested a structure in which PC is first transferred to the antennary GlcNAc that is added by GlcNAc transferase I to the 3-linked mannose of the tri-mannosyl core with additional PC groups being added as further oligosaccharide processing results in other antennary GlcNAc residues being added (Haslam et al. 1997).

In terms of the characterization of glycosylation in nematodes, the majority of publications analyze released glycans from nematode homogenates or excretory–secretory (ES) products. In addition, studies have utilized lectin affinity chromatography to purify glycoproteins from *Caenorhabditis elegans*, which after proteolytic digestion, were then either identified by Edman degradation protein sequencing (Hirabayashi et al. 2002), or after enzymatic removal of N-glycans by mass spectrometry based proteomics (Fan et al. 2005). There have been very few site-specific glycoproteomic characterizations of individual nematode glycoproteins. However, Borloo *et al*. (2013) did characterize *N-*glycosylation sites in the ES glycoproteins of *Cooperia oncophora*. The amino acid sequence of ES-62 contains four potential N-glycosylation sequons with an addition sequon being present in the predicted signal peptide and therefore not present on the mature glycoprotein (Harnett et al. 1999). In the present study, we have employed LC-ES-MS/MS glycoproteomic methodologies to further investigate the structure of ES-62’s PC-containing glycans and also to determine the occupancy and degree of heterogeneity of PC-containing glycans at the different glycosylation sites.

## Results

Samples of the purified ES-62 were subjected to a panel of enzymatic and chemical treatments, designed to characterize the occupancy and glycoforms present at each putative N-glycosylation site. Discounting the 19 residue predicted signal peptide, over 96% of the mature protein was successfully mapped across the tryptic, chymotryptic and Glu-C proteolytic digests, including all four of the N-glycosylation consensus sequences identified with searches targeting previously identified ES-62 glycans (Haslam et al. 1997) (Supplementary Figure 1, Supplementary Table S1).

### Site occupancy

An indication of N-glycosylation site occupancy was achieved by taking aliquots of the tryptic, chymotryptic and Glu-C digested ES-62 and treating each with PNGase-F to remove N-linked glycans and deamidate occupied asparagine residues prior to subsequent analysis by LC-MS^e^. In a MS^e^ experiment data are acquired via alternating between low- and high-collision energy conditions (Silva et al. 2005). However, it should be noted that spontaneous chemical deamidation is also possible (Palmisano et al. 2012; Stephenson and Clarke, 1989). Extracted ion chromatograms (XICs) were then produced from the resultant data, comparing the integrated peak areas for the deamidated (occupied) glycopeptides and their corresponding amidated (non-occupied) versions (Supplementary Figures 2–5). These data demonstrate 100% site occupancy at N254 and N344, with 91% at N213 and 45% at N400.

### PC detection

The detection of putative PC-modified glycopeptides was enabled by exploiting both the marker ions of choline (m/z 104.1) and PC (m/z 184.1) (Figure 1), an approach published by Timm et al. (2015).

The MS^e^ channel (a non-discriminatory method of fragmentation) of each ES-62 digest mass spectral analysis was interrogated for the presence of these marker ions, with the resultant XICs shown in Figure 2. Within each of the digests, there appeared to be a significant number of high-intensity marker ion signals denoting thelabile-fragmentation of the PC groups from their respective glycans. This proved to be a highly effective strategy and allowed the location and characterization of PC-containing glycopeptides, generating a wealth of data on the glycoforms present at each glycosylation site.

**Fig. 4:**
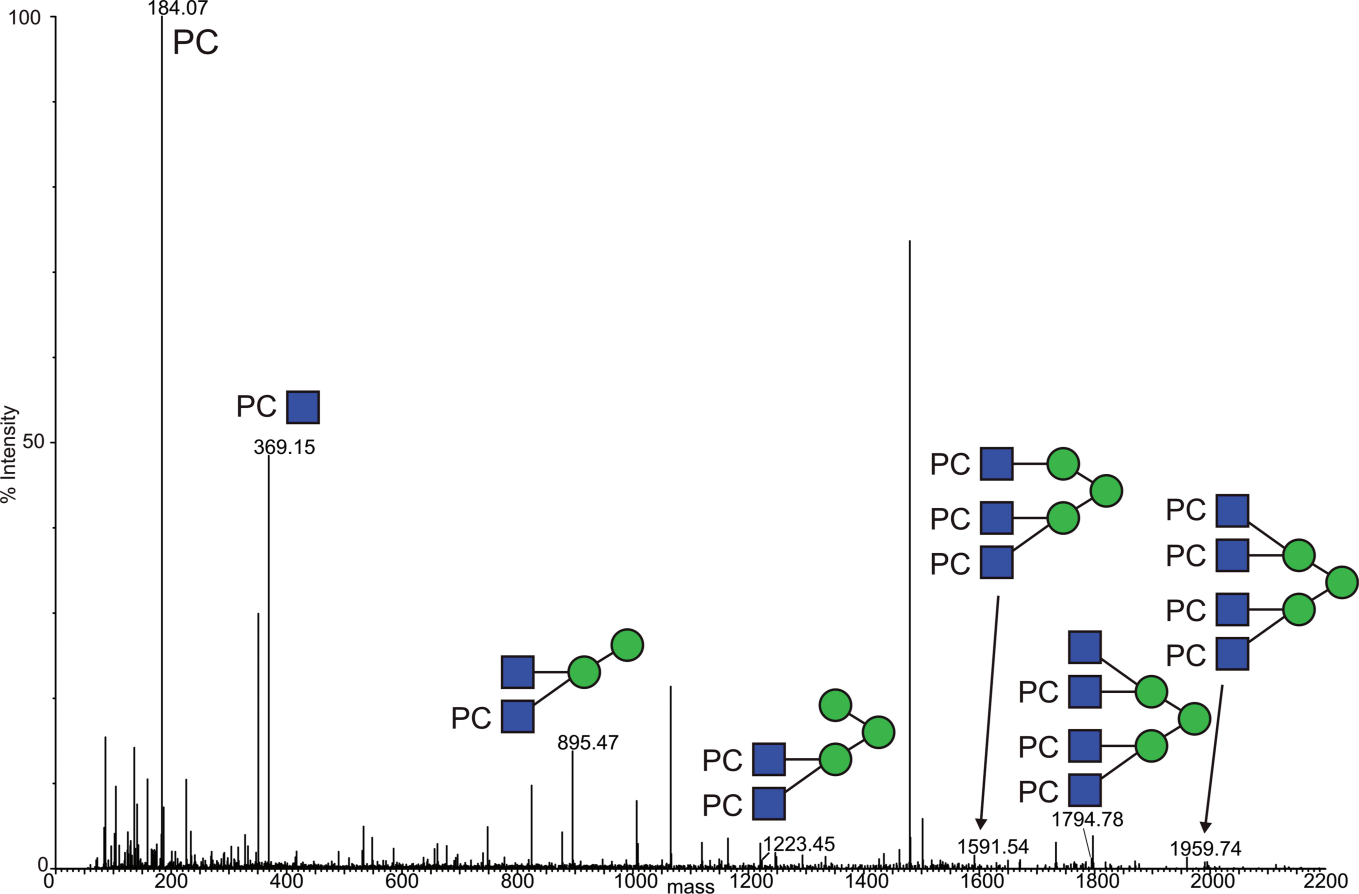
Deconvoluted MS/MS (MS^e^) spectrum of the m/z 5989.53 Asn 213 glycopeptide molecular ion, observed at a retention time of 55.8 min in the reduced, carbamidomethylated trypsin digest of ES-62. Focus is on the low-mass region, showing the fragmented glycan structures. This figure is available in black and white in print and in colour at Glycobiology online.

**Fig. 5:**
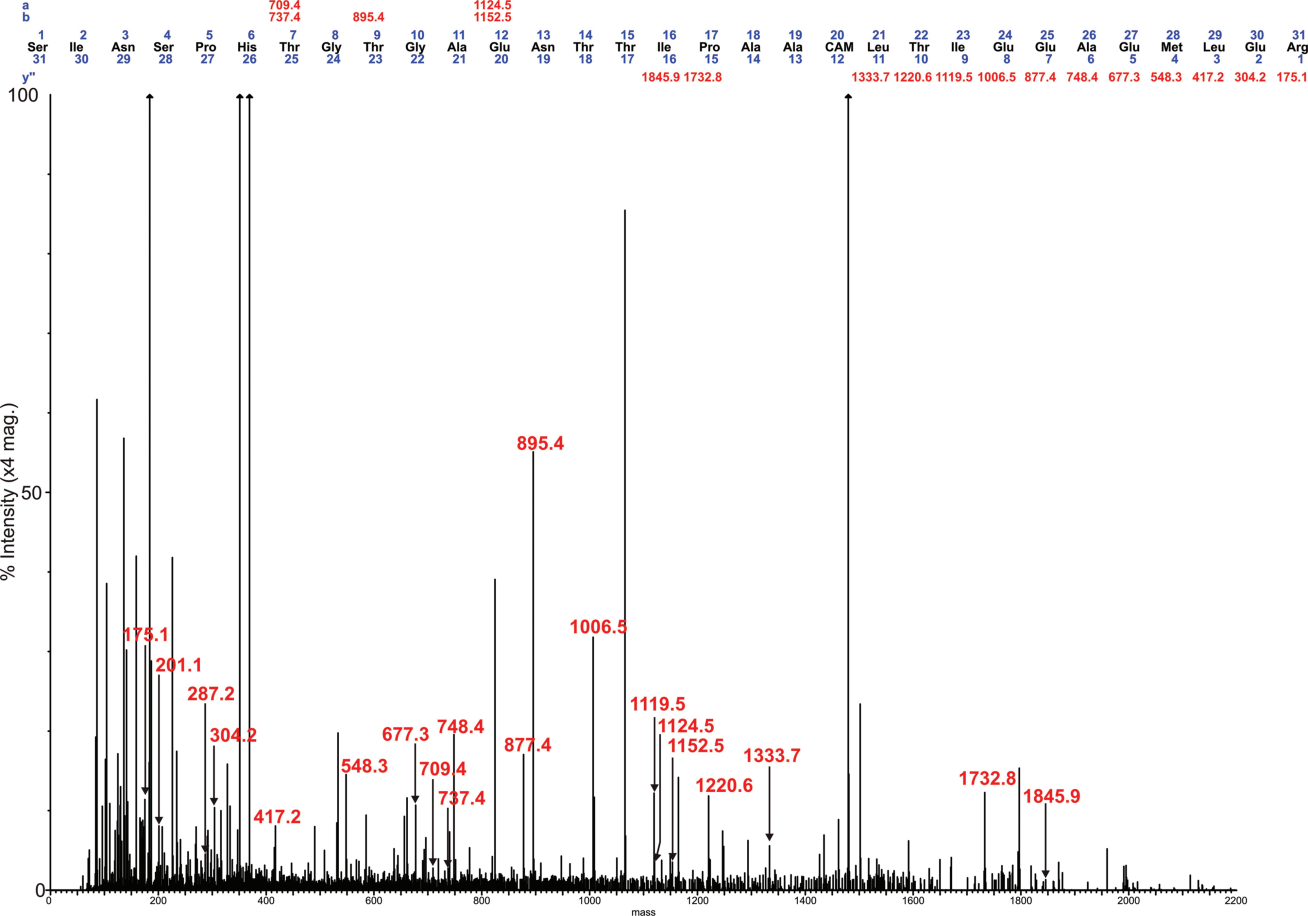
Deconvoluted MS/MS (MS^e^) spectrum of the m/z 5989.53 Asn 213 glycopeptide molecular ion, observed at a retention time of 55.8 min in the reduced, carbamidomethylated trypsin digest of ES-62. Focus is on the low-mass region, showing the peptide fragment ions. This figure is available in black and white in print and in colour at Glycobiology online.

An example of the data quality generated from the fragmentation of PC-modified glycopeptides is shown in Figures 3–5. This selection of data shows the fragmentation of one of the glycopeptides observed at a retention time of 55.8 min in the analysis of the trypsin digest of ES-62. Figure 3 shows the high-mass region of the MS^e^ spectrum, illustrating the sequential loss of PC and glycan residues from the molecular ion at m/z 5988.61. The PC moieties cleave away from the remaining molecule in alternating fashion together with the GlcNAc residues that carry them. In this instance a total of four PC-GlcNAc antennal pairs are removed (together with the core fucose), before the loss of the core mannose residues. Interestingly at this point, a final PC-GlcNAc pair is lost from the core—demonstrating the modification of the distal core GlcNAc, followed by the final proximal GlcNAc to leave the exposed carbamidomethyl-modified tryptic glycopeptide 201–231 at *m/z* 3313.49 (Figure 3). A key fragment ion series is also seen at *m/z* 3313.49, 3516.49, 3719.49 and 3885.69 again indicating the presence of PC modification of the distal core GlcNAc.

The low-mass region of the same spectrum provides further evidence for the structure of the PC-glycan, with a series of peaks corresponding to PC-containing fragments. This region is dominated by the intense PC and PC-GlcNAc signals at m/z 184.07 and 369.15, respectively, together with additional PC-GlcNAc containing fragment ions (Figure 4). No fragment ions at *m/z* 328 (PC-Man) or *m/z* 312 (PC-Fuc) could be observed again providing evidence that PC is only present on GlcNAc residues. It is unusual to observe such an abundance of fragmented glycan data in these analyses across the entire mass range of the spectra, leading to the notion that the PC groups are helping to retain charge and assist in the assignment of the spectra.

The final view of the spectrum (Figure 5) is again the low-mass region, this time magnified to show the confirmation of the peptide sequence, via b and y” peptide fragment ions, also present with the abundant PC-glycan fragment ions. A good proportion of the C-terminus of the glycopeptide is assignable, with a series of clear y” ions apparent before the signals diminish close to the consensus sequence asparagine at residue 213. Additional assigned deconvoluted spectra, which are fully consistent with the above glycan structural features, are displayed in Supplementary Figures 10–12 .

A similar experimental strategy was utilized to characterize all of the N-glycosylation sites. Tables 1 and 2 summarize the proposed individual glycoforms observed at each of the four consensus sites present in the mature protein, using the backbones of the structures previously defined to inform the assignment of fucose, mannose and GlcNAc. The sites are modified by a mixture of bi-, tri- and tetra-antennary truncated glycans, with the Asn400 site additionally modified by high-mannose-type glycans, as previously reported (Haslam et al. 1997). However, while prior analyses have established the potential presence of a single PC on the multiantennary complex structures, these data clearly demonstrate the extent to which this modification is present, with up to five additions on the tetra-antennary glycans.

**Table I:** Summary of the glycan structures observed at each consensus site within the ES-62 molecule. The proposed structures of the PC containing N-glycans have not been fully defined, for example in terms of branching pattern for triantennary structures. Structural data from previous characterization of ES-62 was taken into account (Haslam et al. 1997).

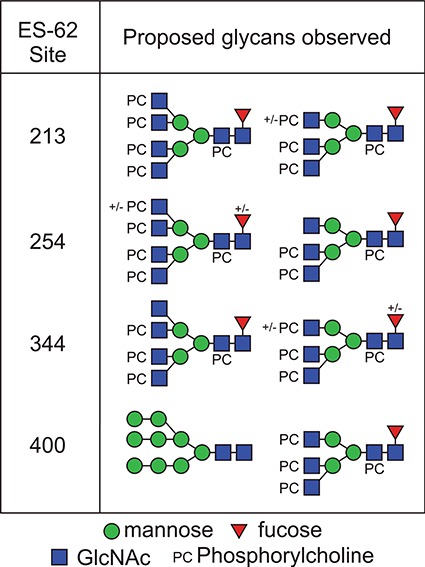

**Table II:** Summary of the LC-MS data, showing glycopeptides representing all of the unique glycoforms observed at each site within the analyses of the three digests.

**Sample preparation**	**N-linked site**	**Peptide sequence**	**Retention time (min)**	**Deconvoluted observed glycopeptide mass (Da)**	**Calculated peptide mass (Da)**	**Calculated glycan mass (Da)**	**Proposed glycan structure**
Chymotrypsin (RCAM)	213	SINSPHTGTGAENTTIPAAC_AM_ L	42.6	4419.83	2111.00	2309.85	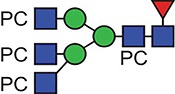
Chymotrypsin (RCAM)	213	SINSPHTGTGAENTTIPAAC_AM_ L	43.0	4254.75	2111.00	2144.80	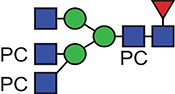
Trypsin (RCAM)	213	SINSPHTGTGAENTTIPAAC_AM_ LTIEEAEMLER	55.8	5989.50	3312.56	2677.99	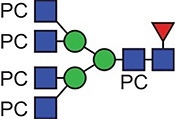
Trypsin (RCAM)	213	SINSPHTGTGAENTTIPAAC_AM_ LTIEEAEMLER	55.8	5621.45	3312.56	2309.85	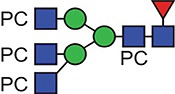
Chymotrypsin (RCAM)	254	DMKSHYEEPINSSNL	39.2	4274.71	1762.78	2512.93	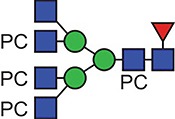
Chymotrypsin (RCAM)	254	DMKSHYEEPINSSNL	39.4	3906.58	1762.78	2144.80	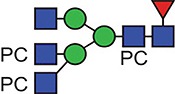
Chymotrypsin (RCAM)	254	DMKSHYEEPINSSNL	39.7	4294.71	1762.78	2531.93	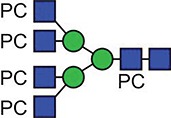
Chymotrypsin (RCAM)	254	DMKSHYEEPINSSNL	39.8	4128.64	1762.78	2366.87	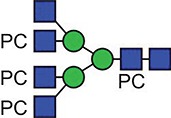
Glu-C (RCAM)	344	QGYGGAKHYYITHKNDSP	46.5	4548.84	2034.95	2512.93	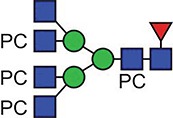
Glu-C (RCAM)	344	QGYGGAKHYYITHKNDSP	46.7	4345.75	2034.95	2309.85	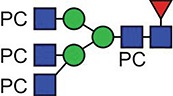
Glu-C (RCAM)	344	QGYGGAKHYYITHKNDSP	47.0	4199.69	2034.95	2163.79	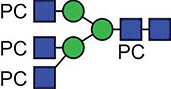
Glu-C (RCAM)	344	QGYGGAKHYYITHKNDSP	47.2	4034.65	2034.95	1998.74	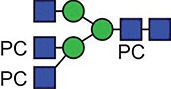
Glu-C (RCAM)	400	ITRLLSRNGIALGLINSSVQGD	52.9	4162.87	2296.29	1866.65	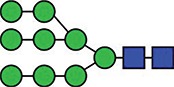
Glu-C (RCAM)	400	ITRLLSRNGIALGLINSSVQGDVTFWAK	52.9	5340.23	3028.68	2309.85	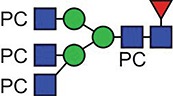

The proposed structures of the PC containing N-glycans have not been fully defined, for example, in terms of branching pattern for triantennary structures.

Structural data from previous characterization of ES-62 was taken into account (Haslam et al. 1997).

As a consequence of the quality of the PC-glycopeptide data described above we hypothesize that the presence of the PC residues has a beneficial effect on the fragmentation spectra. To investigate this an aliquot of the digested samples was treated with hydrofluoric acid (HF) in order to break the phosphodiester bonds and remove the PC moieties from the glycans. These samples were then once more analyzed by LC-MS and the data quality of the glycopeptide fragmentation compared to that of the non-treated samples. Partial removal of the PC residues was achieved (Supplementary Figures 6–9), with a commensurate reduction in the quality of the MS and MS^e^ data, rendering assignment of the fragmentation spectra much more challenging. This provides some supporting evidence that the quality of the fragment ion data observed in the MS/MS spectra of the glycopeptides in this study is linked to the presence of the PC-moieties. However, from the data presented it cannot be ruled out that the HF treatment has caused additional degradation of the peptide backbone.

From an analytical perspective we observed that the presence of the PC-functional group on N-glycopeptides greatly enhanced their sensitivity of detection by ES-MS. We presume this is due to the additional permanent positive charge of the choline in the PC group. Therefore, addition of PC groups onto other glycopeptides could enhance their detection and characterization.

## Discussion

Recently, there has been much interest in developing parasitic worm-derived molecules as therapeutics for the treatment of a range of human ailments associated with aberrant inflammatory responses (reviewed by Harnett and Harnett 2017). ES-62 is generally considered to be in the forefront of this development and so there is a need to learn as much as possible about its biology. Of particular relevance is its PC moiety as this appears to be essentially responsible for its anti-inflammatory activities (reviewed by Pineda et al. 2014) and constitutes the starting point for an SMA drug discovery program (Al-Riyami et al. 2013). In this latest study focusing on ES-62, we have undertaken, to our knowledge, the most detailed glycoproteomic characterization of a nematode glycoprotein to date and in the process have demonstrated that all four potential *N-*glycosylation sites on the mature glycoprotein are occupied by PC-containing *N*-glycans.

Our original studies on the structure of ES-62’s PC-glycan suggested that PC was transferred to antennary GlcNAc residues, during the process of the latter being added to the glycan during oligosaccharide processing (reviewed by Harnett, Rzepecka and Houston (Harnett et al. 2010)). We were subsequently able to confirm the existence of the same structures in other filarial nematode species including the medically important human pathogen that causes river blindness, *Onchocerca volvulus* (Haslam et al. 1999). Furthermore, around the same time PC was found attached to GlcNAc residues on nematode glycosphingolipids (Lochnit et al. 1998). Subsequently identical PC-sugar structures and in some cases additional PC-*N*-glycan structures have been found in a number of nematode species (reviewed by Hokke and van Diepen 2017) including the free-living *C. elegans* (reviewed by Cipollo et al. 2004; Haslam et al. 2002). In our original work on the PC-*N*-glycans of filarial nematode species (Haslam et al. 1999; Haslam et al. 1997), we presented evidence for the existence of up to two PC moieties per glycan. However, in this new study it is clear that ES-62 *N*-glycans may contain up to five PC groups. FAB-MS- and MALDI-MS-based structural characterization of released PC-containing *N*-glycans is very challenging due to the zwitterionic nature of the PC functional group and potential difficulty in releasing heavily PC-substituted N-glycans with enzymes such as peptide N-glycosidase F (PNGase F) and PNGase A: this could be especially an issue for PC-modified cores. However, in this study ES-MS-based analysis of PC containing *N*-glycopeptides is better able to handle the multiple potential charge states, thus increasing the sensitivity of detection of the higher PC substituted *N*-glycans. Four of the PC groups are proposed to be on antennary GlcNAc residues with the fifth on the distal core GlcNAc. The latter substitution was not detected in our earlier work but has previously been described with respect to *C. elegans* (Cipollo et al. 2005), although the indication was that PC could be added to both distal and proximal core GlcNAc in the *N*-glycans of this species.

The identity of the enzyme that transfers PC to carbohydrates in nematodes is unknown, but we have previously speculated (Harnett et al. 2010) on whether the same or different transferases might deliver PC to GlcNAc on both *N*-glycans and glycosphingolipids. Support in favor of the former is perhaps that glycosyltransferases have been reported to target both structures in vitro (reviewed by Paulson and Colley 1989) and also that the acceptor for PC is the same for both classes of molecule, the C-6 position of GlcNAc (Gerdt et al. 1999; Haslam et al. 2002). If the one-PC transferase model is indeed the case, then the same enzyme should also transfer PC to core GlcNAc in *N*-glycans, and hence it would appear that this sugar is simply the structure required to act as a PC acceptor. However, the same glycosyltransferase is generally not employed for modifying core and antenna sugars, and the fact that we do not observe any PC modification of the core GlcNAc residues of the high-mannose glycans observed at the Asn400 site would seem to indicate a degree of structural preference for the PC transferase. Furthermore, PC has been found attached to terminal *N*-acetylgalactosamine in the porcine nematode *Trichuris suis* (Wilson and Paschinger 2016), indicating that if only one enzyme exists, it might be capable of transferring PC to more than one sugar. In addition, in that study, although N-glycans with terminal GlcNAc were present, PC was not found attached to terminal GlcNAc but was found attached to sub-terminal GlcNAc, raising the possibility that *T. suis* may perhaps possess a different PC transferase to that found in those species that transfer to terminal GlcNAc. Vanbeselaere et al. (2018) also recently described an unusual PC-substituted glycosaminoglycan-like O-glycan from the parasitic nematode *Oesophagostomum dentatum* in which PC groups were determined on both HexNAc and galactose residues. Overall, therefore, the question as to how many different PC transferases nematodes contain remains to be answered, and it may be that the answer is different for individual species.

Our previous work in characterizing ES-62’s *N*-glycans also revealed the existence of other types of structure in particular high-mannose glycans and also glycans fully trimmed to the tri-mannosyl core with or without core fucose (Haslam et al. 1997). This raised the question as to how many of ES-62’s potential *N*-glycosylation sites actually contain PC-glycans, but the answer from the present glycoproteomics analysis of ES-62 indicates it is all four. As most of the PC, being attached to terminal GlcNAc, at the end of glycan chains, this raises the possibility that ES-62 as a tetrameric molecule of molecular mass ~240 kDa presents a substantial PC “coat” to the external environment, and such PC will have ample opportunity to interact with the molecules and cells of the immune system. Such molecules include antibodies and C-reactive protein. Antibodies in theory could remove ES-62 from the circulation but our previous studies in the model jird system suggest that this only happens in the early stages of antibody production and not during a chronic infection, a finding that may reflect a reduction in the size of immune complexes as the antibody response to ES-62 moves from IgM to IgG (smaller immune complexes tend to persist longer) (Harnett et al. 1990). In any case, by presenting so many PC groups to the external environment, it is possible that not all ES-62 PC groups are bound by antibodies such that the potential for direct interaction with receptors on immune system cells remains. Nevertheless, antibodies may actually enhance uptake of ES-62 by Fc receptor-expressing immune system cells, and it is possible that under such circumstances the molecule may remain functional. Regarding C-reactive protein, studies employing human serum reveal that this is in fact the major protein bound by ES-62 with extremely large complexes forming. Such interaction with C-reactive protein is of high affinity and results in blockage of the molecule’s ability to activate complement (Ahmed et al. 2016). Once again, the large number of PC groups presented by ES-62 will be likely to contribute to what is a very effective inhibitory effect. In addition, it should facilitate multiple engagements with surface receptors of immune system cells, which may help explain its potent ability to modulate their activities (reviewed by Pineda *et al.* 2014). Based on our site-specific glycoproteomic analysis, any one ES-62 tetramer (the form in which the molecule is secreted) can contain up to 72 PC groups, the majority of which, being present on antenna GlcNAc, will point into space. This means that the PC on ES-62 has significant potential to interact with appropriate receptors on cells and molecules of the immune system and this may explain its potent immunomodulatory effects.

## Materials and methods

### ES-62 preparation

Highly purified ES-62 was prepared from spent culture medium of adult mixed-sex *A. viteae* by ultrafiltration as described previously (Wilson et al. 2003).

#### Sample Preparation

Aliquots of the purified ES-62 (~250 μg in 100 μL phosphate-buffered saline) was made up to 253 μL with addition of 153 μL of 50 mM ammonium bicarbonate (ambic) buffer, pH 8.4.

#### Protein Denaturation

A total of 20 μL of a 0.1%(w/v) solution of RapiGest™ SF Surfactant (Waters, UK) was added to the sample. The sample was incubated at 60°C for 15 min.

#### Reduction and Alkylation

Reduction of thiol bridges and free thiols was carried out by addition of 5 μL of a 20 mg/mL solution of dithiothreitol (DTT) in 50 mM ambic buffer, pH 4.8, followed by incubation at 60°C for 60 min. Alkylation of exposed residues was carried out by addition of 12 μL of a 20 mg/mL solution of iodoacetamide in 50 mM ambic buffer, pH 8.4, followed by incubation at ambient temperature for 30 min in the dark. Quenching of excess alkylation agent was performed by the addition of 10 μL of a 20 mg/mL solution of DTT in 50 mM ambic buffer, pH 8.4, followed by incubation at ambient temperature for 10 min. The sample was split into three identical aliquots of 100 μL each for proteolytic digestion.

#### Chymotryptic Digest

A total of 100 μL of the reduced and alkylated ES-62 sample was digested by adding 10 μL of a 1 mg/mL solution of chymotrypsin in 50 mM ambic buffer, pH 8.4, followed by incubation at 37°C for 18 h.

#### Endoproteinase (Glu-C) Digest

A total of 100 μL of the reduced and alkylated ES-62 sample was digested by adding 10 μL of a 1 mg/mL solution of Glu-C in 50 mM ambic buffer, pH 8.4, followed by incubation at 37°C for 18 h.

#### Tryptic Digest

A total of 100 μL of the reduced and alkylated ES-62 sample was digested by adding 10 μL of a 1 mg/mL solution of trypsin in 50 mM ambic buffer, pH 8.4, followed by incubation at 37°C for 18 h.

#### RapiGest™ SF Surfactant Precipitation

Acid-labile surfactant precipitation was carried out by the addition of 0.5 μL of trifluoroacetic acid to each of the three digested samples. Each sample was incubated at 37°C for 45 min, followed by centrifugation at 150,000 × *g* for 15 min. The supernatant from each sample was recovered and transferred to fresh tubes.

#### PNGase F Digestion

An aliquot of each digested sample (corresponding to ~25 μg of digested material) was taken and lyophilized. Each freeze-dried digest sample was resuspended in 25 uL of 50 mM ambic buffer, pH 8.4. A total of 2 U of PNGase F was added and incubated for 24 h (with an additional 2 U aliquot of PNGase F added after 6 h).

#### HF Cleavage of Phosphodiester Bonds

An aliquot of each digested sample (corresponding to ~25 μg of digested material) was lyophilized. Each freeze-dried digest sample was resuspended in 50 uL of 48% HF and incubated on ice for 20 h. The reaction was terminated by gentle drying under nitrogen, followed by resuspension in UHQ H_2_O and lyophilization.

#### Nano-LC MS and MS/MS Analysis of (glyco)peptides

Sample digests, resuspended in 0.1% (v/v) formic acid, were analyzed by on-line nano-flow reverse-phase high-performance liquid chromatography with online electrospray-mass spectrometric analysis (nano-RP-HPLC-ES-MS) with MS/MS (MS^e^) using a Waters SYNAPT G2-S high-definition mass spectrometer, coupled to a Waters ACQUITY UPLC M-Class System (Waters UK, Elstree). Separations were achieved by means of a C18 trapping column (M-Class Symmetry C18 Trap, 100 Å, 5 μm, 180 μm × 20 mm, 2G) connected in-line with a 75 μm C18 reverse-phase analytical column (M-Class Peptide BEH C18, 130 Å, 1.7 μm, 75 μm × 150 mm) eluted over 90 min with a gradient of acetonitrile in 0.1% formic acid at a flow rate of 300 nL/min. Column temperatures were maintained at 50°C, and data were recorded in MS^e^ “Resolution” positive ion mode, with scan times set to 0.5 s in both the high-energy and low-energy modes of operation. The instrument was pre-calibrated using 10–100 fmol/μL of [Glu^1^]-fibrinopeptide B/5% (v/v) acetic acid (1:3, v/v) and calibrated during analysis by means of a lockmass system using [Glu^1^]-Fibrinopeptide B 785.8426^2+^ ion. The collision gas utilized was argon with collision energy ramp of 20–45 eV. Data acquisition was performed using MassLynx (Waters UK, Elstree) software and analyzed by means of MassLynx, BiopharmaLynx and ProteinLynx Global Server (PLGS) version 3.0.2 (Waters UK, Elstree).

## Supplementary Material

Supplementary_Figures_v2_cwz035

Supplementary_Tables_cwz035

## Abbreviations

CDNSHP: completely de-sulfated, *N*-sulfated heparin
ECM: extracellular matrix
GAGs,: glycosaminoglycans
Gal,: β-D-galactose
GalNAc: *N*-acetyl-D-galactosamine
GlcA: β-D-glucuronic acid
GlcNAc: *N*-acetyl-α-D-glucosamine
GST: glutathione S-transferase
NAHP: non-anticoagulant heparin
HN: heparosan
HN6S: heparosan 6S
HP: heparin
HS: heparan sulfate
NSHN: *N*-deacetylated, *N*-sulfoheparosan;
NSHN2S: *N*-deacetylated, *N*-sulfated C5-epimerase treated 2-*O*-sulfated heparosan
NSHN6S: *N*-deacetylated, *N*-sulfated, 6-*O*-sulfated heparosan
IdoA: α-L-iduronic acid
IPTG: isopropyl-1-thio-β-D-galactopyranoside
PGs: proteoglycans
UA: uronic acid
