# Supplementary material for: Site-specific glycoproteomic characterization of ES-62: The major secreted product of the parasitic worm Acanthocheilonema viteae
Source: Glycobiology. 2019 May 16;29(8):562–71. doi: 10.1093/glycob/cwz035 (PMC6639541; doi:10.1093/glycob/cwz035)
Supplement: Supplementary_Figures_v2_cwz035 [file supplementary_figures_v2_cwz035.pdf]

1 MLLNSSTFFFLVTLTVVLGAAVLPDKTVAP 30
31 KNYIQETFGKEVAELIQYITKGEEVGLAYQ 60
61 WLSKLVDGFGHRMVGSDSLEKSIAFLEESL 90
91 KNDNFDKVHTEEVPNLPHWVRGNDVVEMIE 120
121 PRNQRLNVLAIGGSEPASATGEVTVIYDLD 150
151 DVKPDDVRGKIVVTAQTFAGYPLTLKYRRS 180
181 VKLFEQLGAIGVLVKSITSFSINSPHTGTG 210
211 AENTTIPAACLTIEEAEMLERLYRSGKKIV 240
241 IRMDMKSHYEEPINSSNLIFEITGSERPSE 270
271 VVLLSAHVDSWDVGQGALDDGAGCAVVWSA 300
301 LHSLKKLAERNPKFKPKRTIRGIFWTSEEQ 330
331 GYGGAKHYYITHKNDSPEKFYFVSETDTGT 360
361 FKSTNWLAHLSFSGDKKSMLRLKEITRLLS 390
391 RNGIALGLINSSVQGDVTFWAKDGIPSVNY 420
421 IPDKAVDYYFYFHHTAGDYMTVLKDGDLEY 450
451 TTSIFATLGHVIANMDDWGSDPNQPQQLNS 480
481 KQSTTEKSDRKKL

Trypsin Chymo Glu-C

Supplementary Figure 1 – Primary amino acid sequence of ES-62. Sequence coverage achieved by means of trypsin (blue underline), chymotrypsin (orange underline) and Glu-C (magenta underline) is indicated, together with overall sequence coverage (grey highlight) and the locations of the potential N-linked consensus sites at residues 213, 254, 344 and 400.

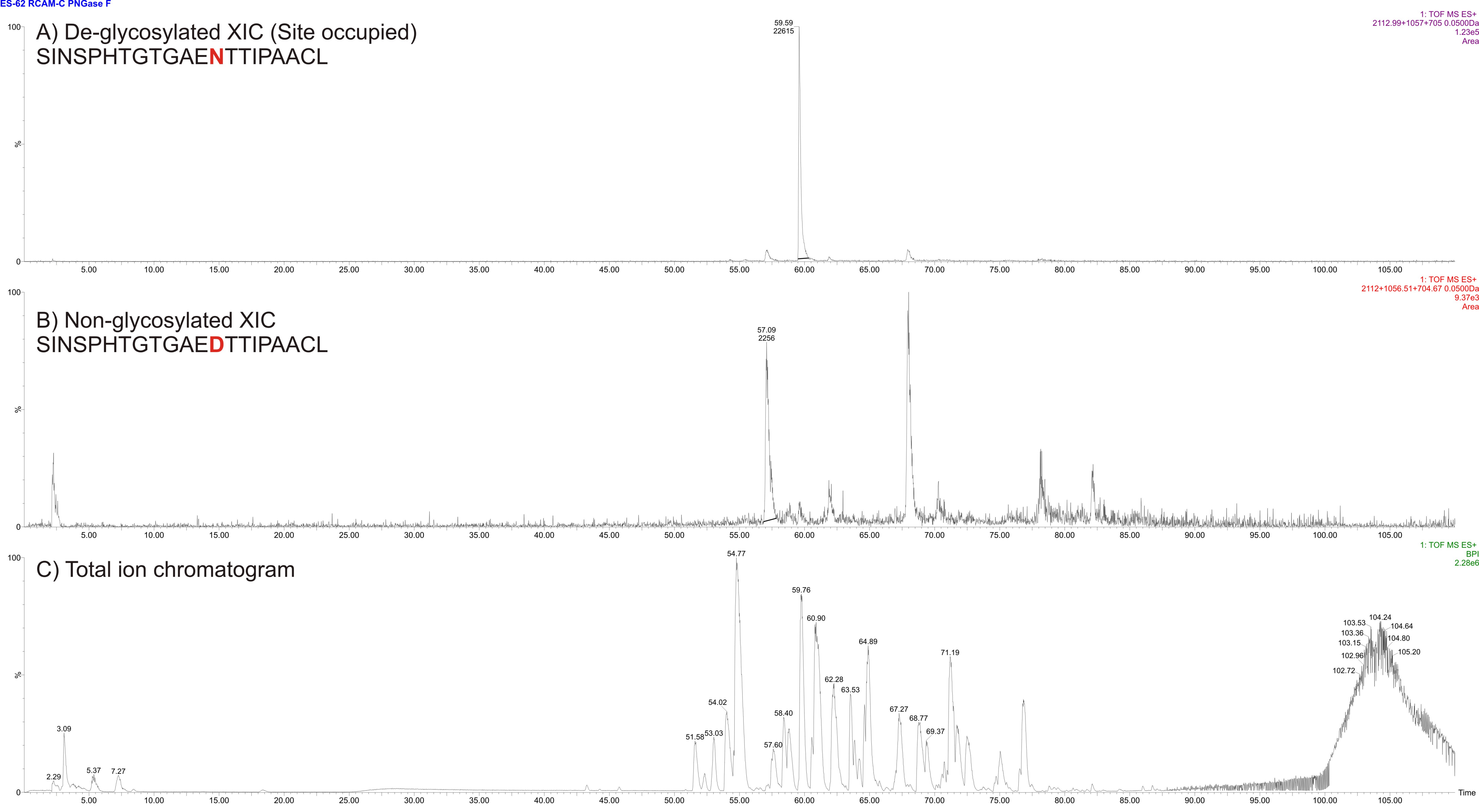

Supplementary Figure 2 - Extracted ion chromatograms from the reduced, carbamidomethylated PNGase-F treated chymotrypsin digest of ES-62, comparing the signals corresponding to an occupied (A) and non-occupied (B) versions of a typical residue 213 containing glycopeptide. Unlabelled peaks contain coincidental masses - usually from incorrect charge states or isotope peaks.

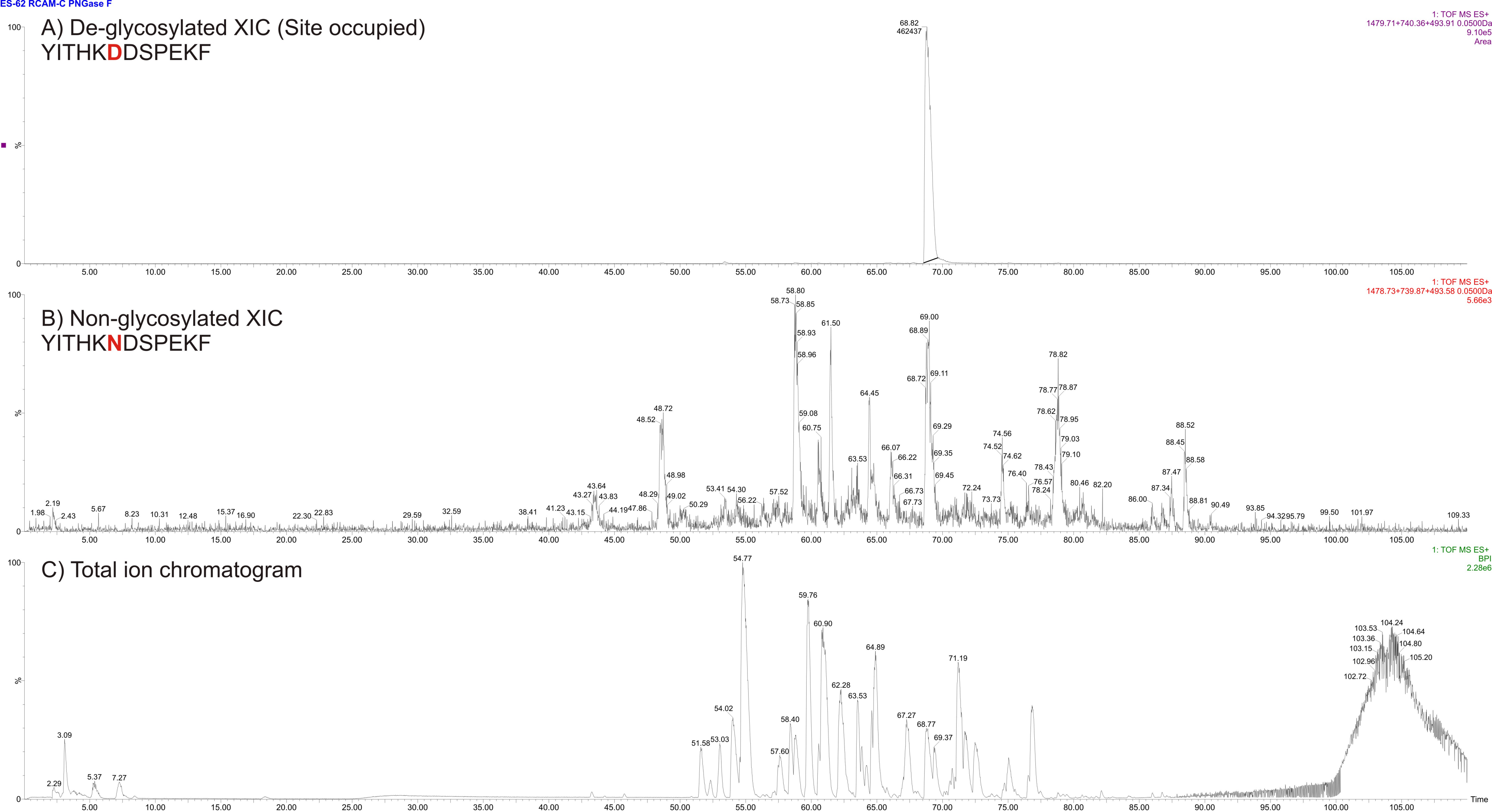

Supplementary Figure 3 - Extracted ion chromatograms from the reduced, carbamidomethylated PNGase-F treated chymotrypsin digest of ES-62, comparing the signals corresponding to an occupied (A) and non-occupied (B) versions of a typical residue 344 containing glycopeptide. Unlabelled peaks contain coincidental masses - usually from incorrect charge states or isotope peaks.

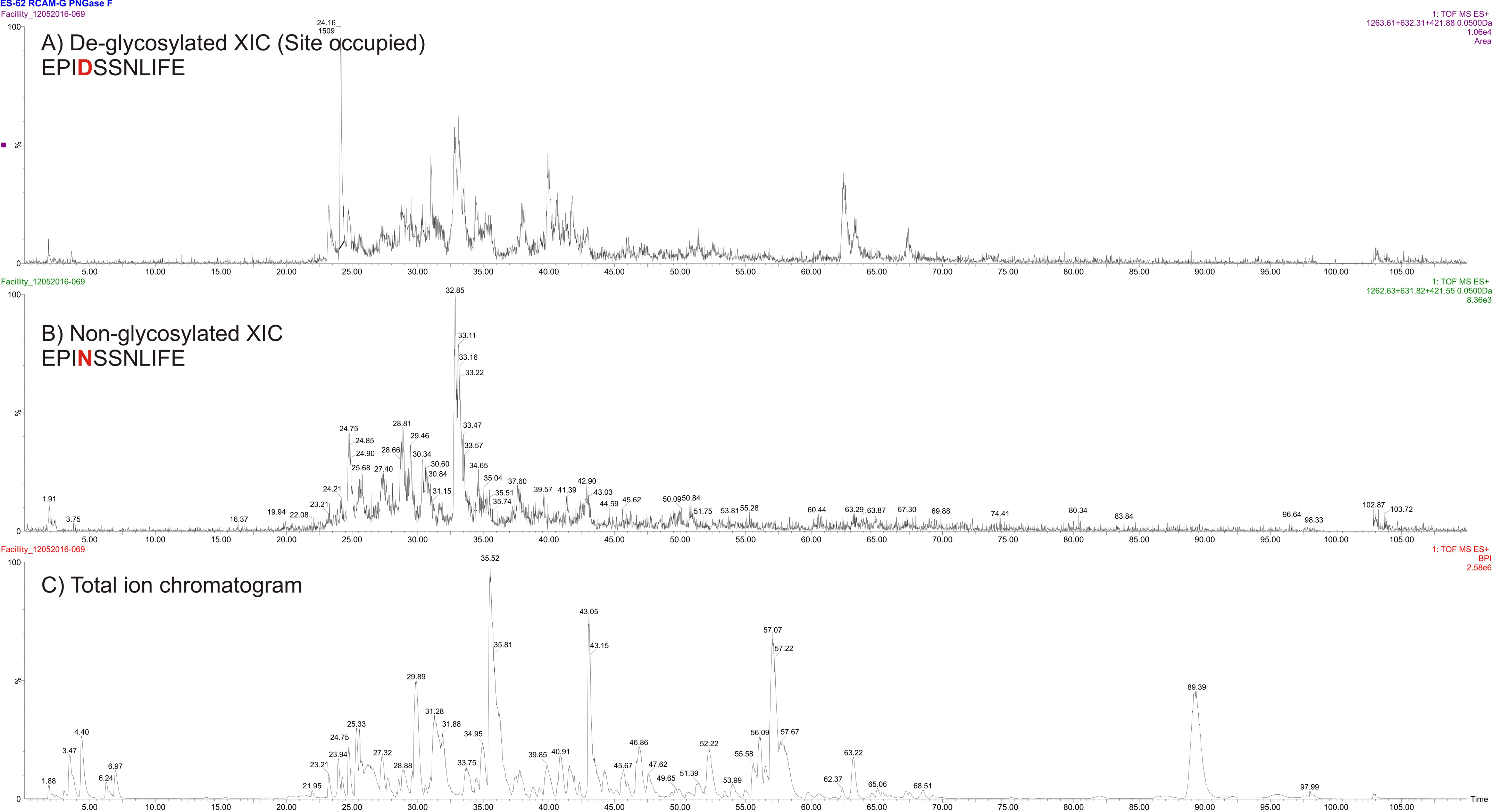

Supplementary Figure 4 - Extracted ion chromatograms from the reduced, carbamidomethylated PNGase-F treated Glu-C digest of ES-62, comparing the signals corresponding to an occupied (A) and non-occupied (B) versions of a typical residue 254 containing glycopeptide. Unlabelled peaks contain coincidental masses - usually from incorrect charge states or isotope peaks.

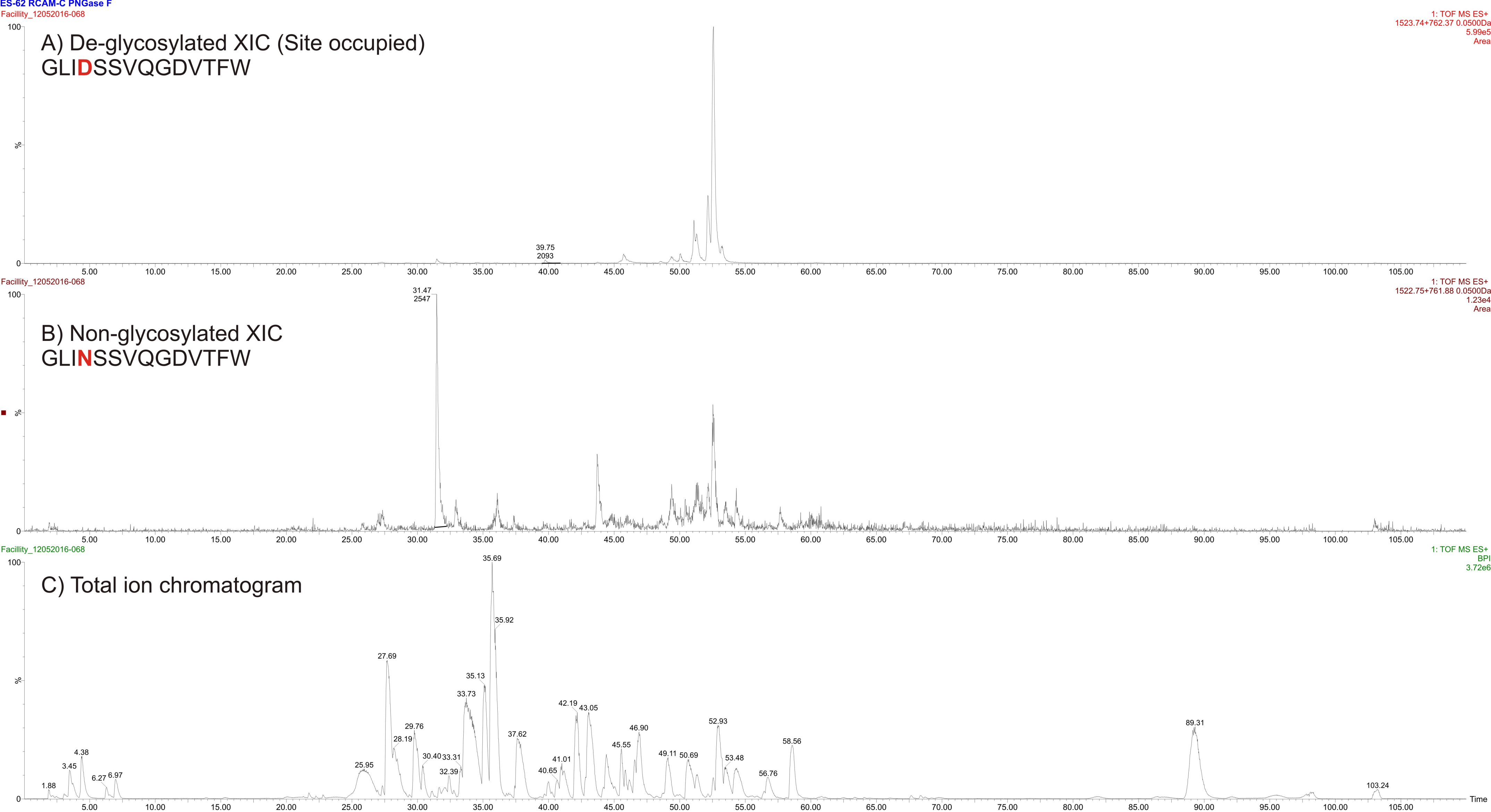

Supplementary Figure 5 - Extracted ion chromatograms from the reduced, carbamidomethylated PNGase-F treated chymotrypsin digest of ES-62, comparing the signals corresponding to an occupied (A) and non-occupied (B) versions of a typical residue 400 containing glycopeptide. Unlabelled peaks contain coincidental masses - usually from incorrect charge states or isotope peaks.

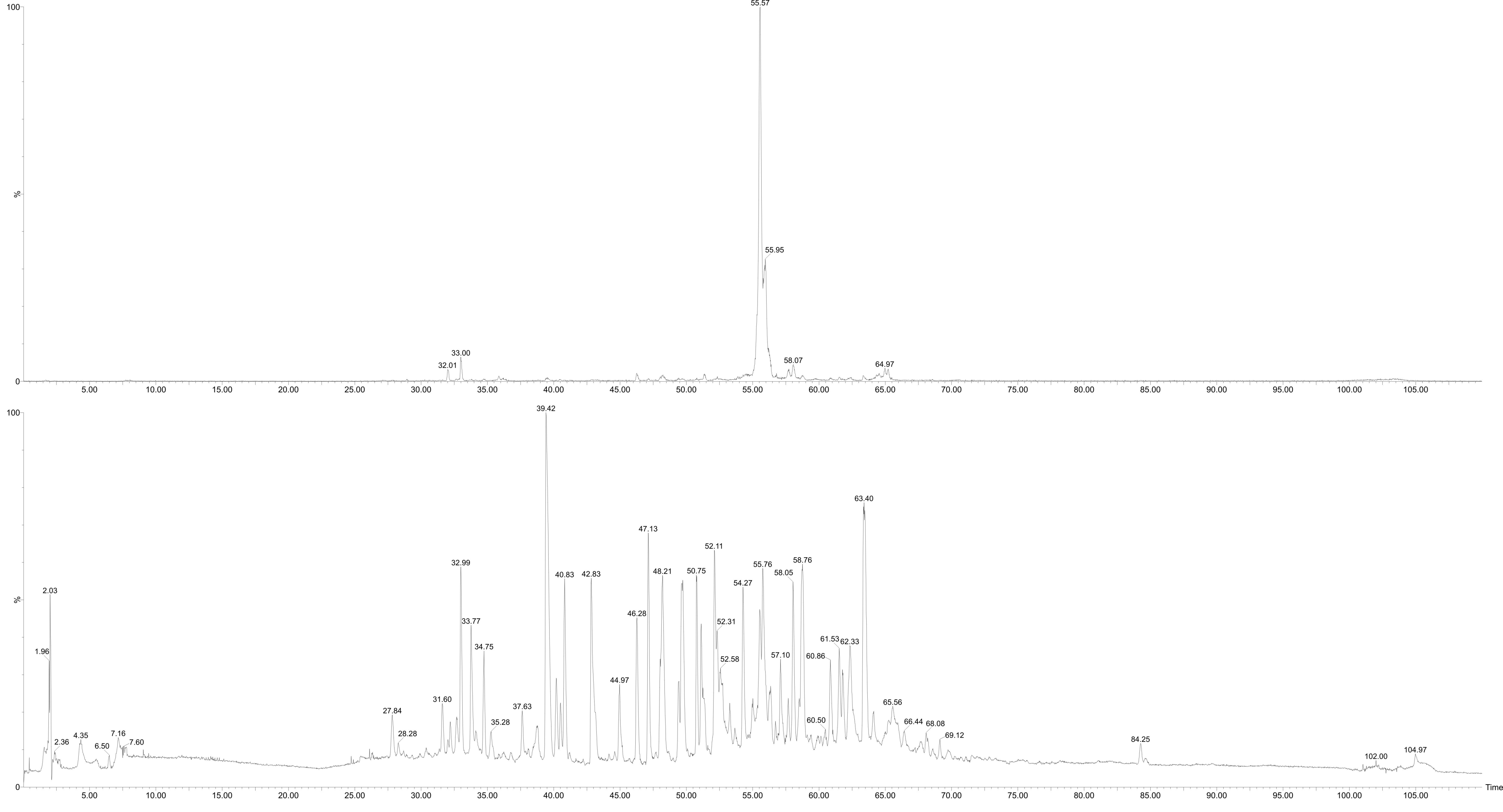

Supplementary Figure 6 - Extracted ion chromatograms from the reduced, carbamidomethylated trypsin digest of ES-62, showing the signals corresponding to PC (m/z 104.1 and 184.1) (top) versus the molecular ion TIC (bottom)

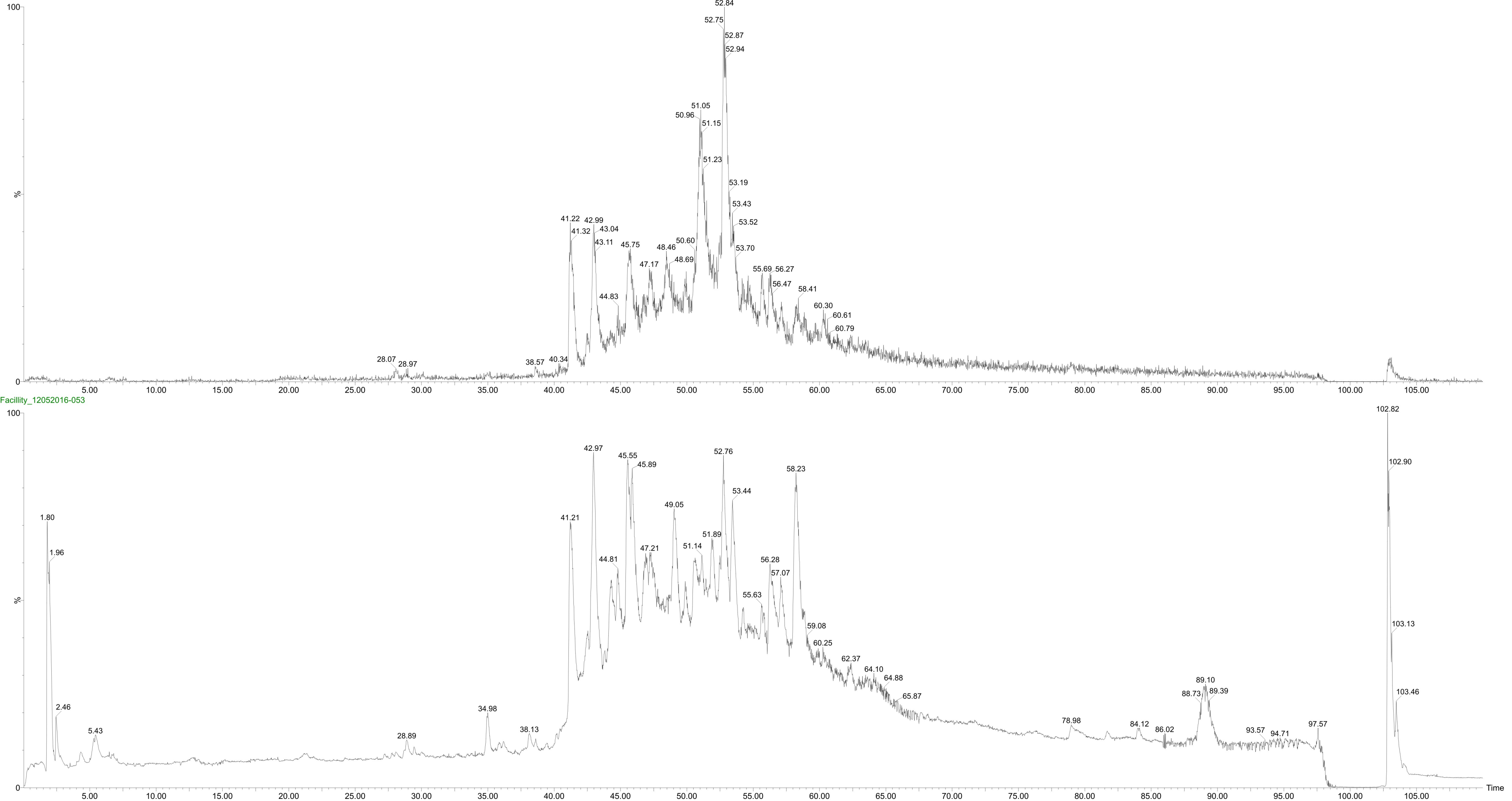

Supplementary Figure 7 - Extracted ion chromatograms from the reduced, carbamidomethylated HF treated trypsin digest of ES-62, showing the signals corresponding to PC (m/z 104.1 and 184.1) (top) versus the molecular ion TIC (bottom) - significantly distorting the native XIC in Supplementary Figure 6

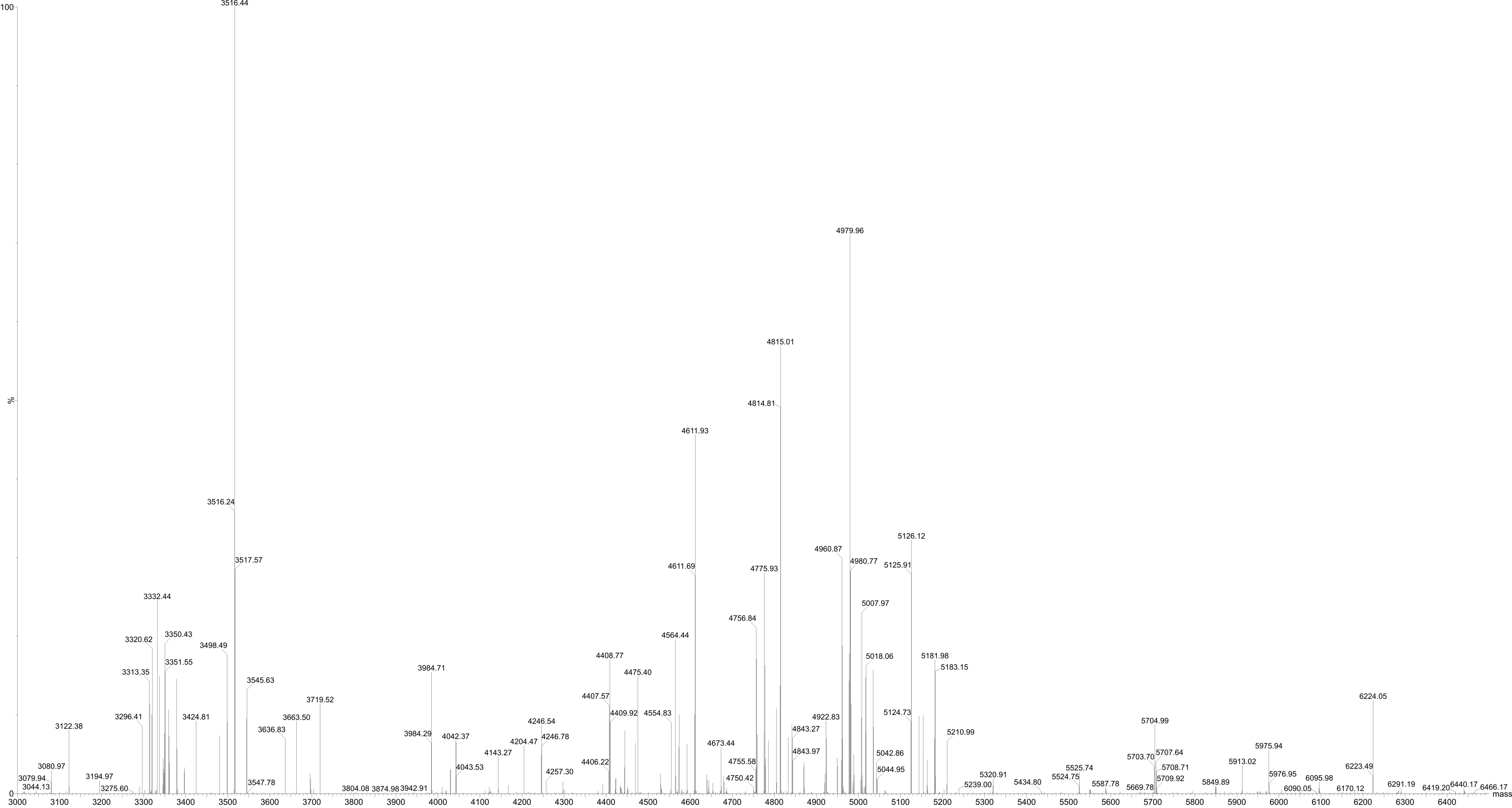

Supplementary Figure 8 - Deconvoluted MS^{e} mass spectrum of the peak corresponding to the non-PC modified FucHexNAc$_4$Hex$_3$ peak from the reduced, carbamidomethylated HF treated trypsin digest of ES-62 shown in Supplementary Figure 7. High mass region, demonstrating loss in quality of the data when PC is absent

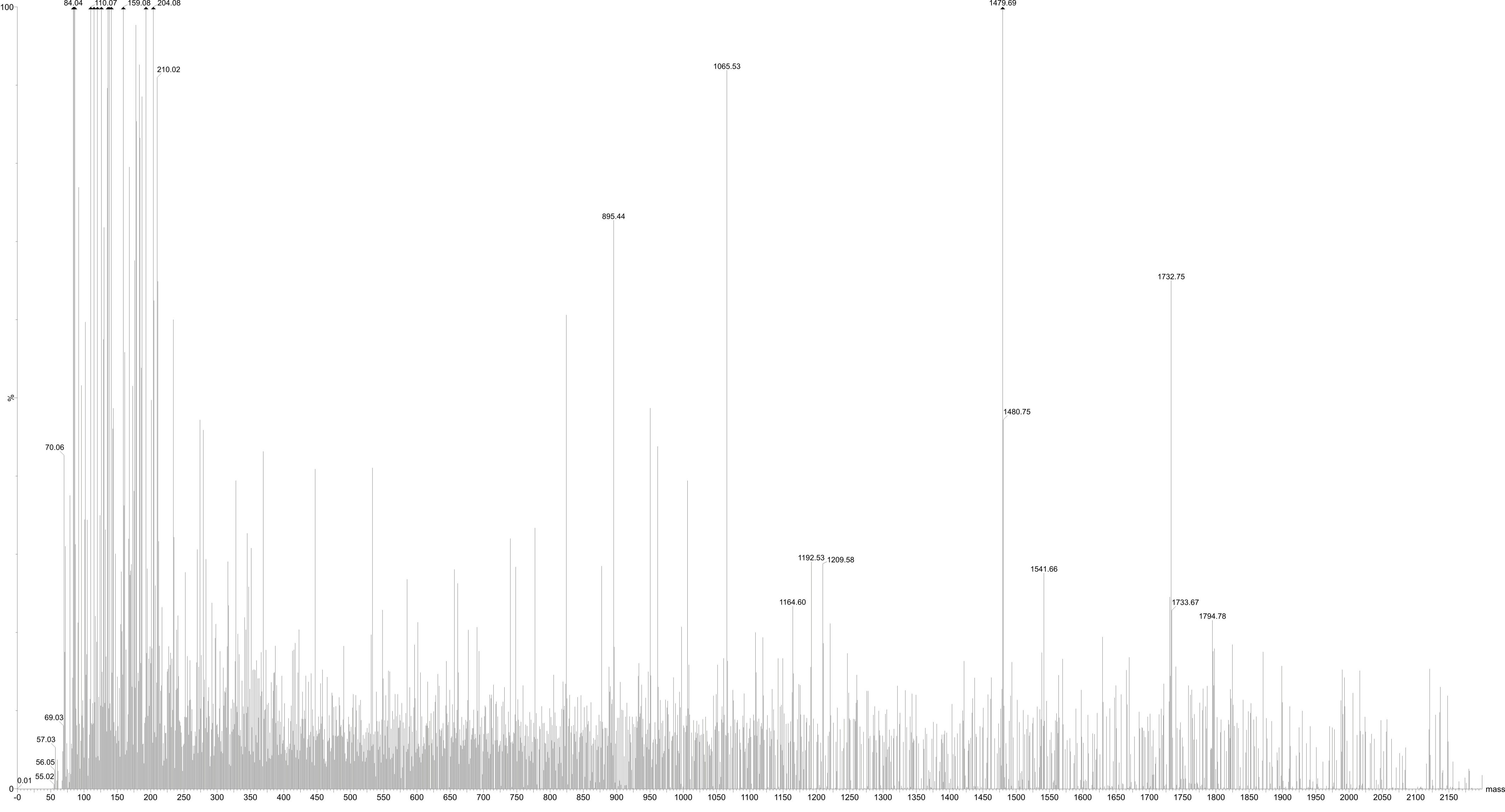

Supplementary Figure 9 - Deconvoluted MS^e mass spectrum of the peak corresponding to the non-PC modified $FucHexNAc_4Hex_3$ peak from the reduced, carbamidomethylated HF treated trypsin digest of ES-62 shown in Supplementary Figure 7. Low mass region, demonstrating loss in quality of the data when PC is absent

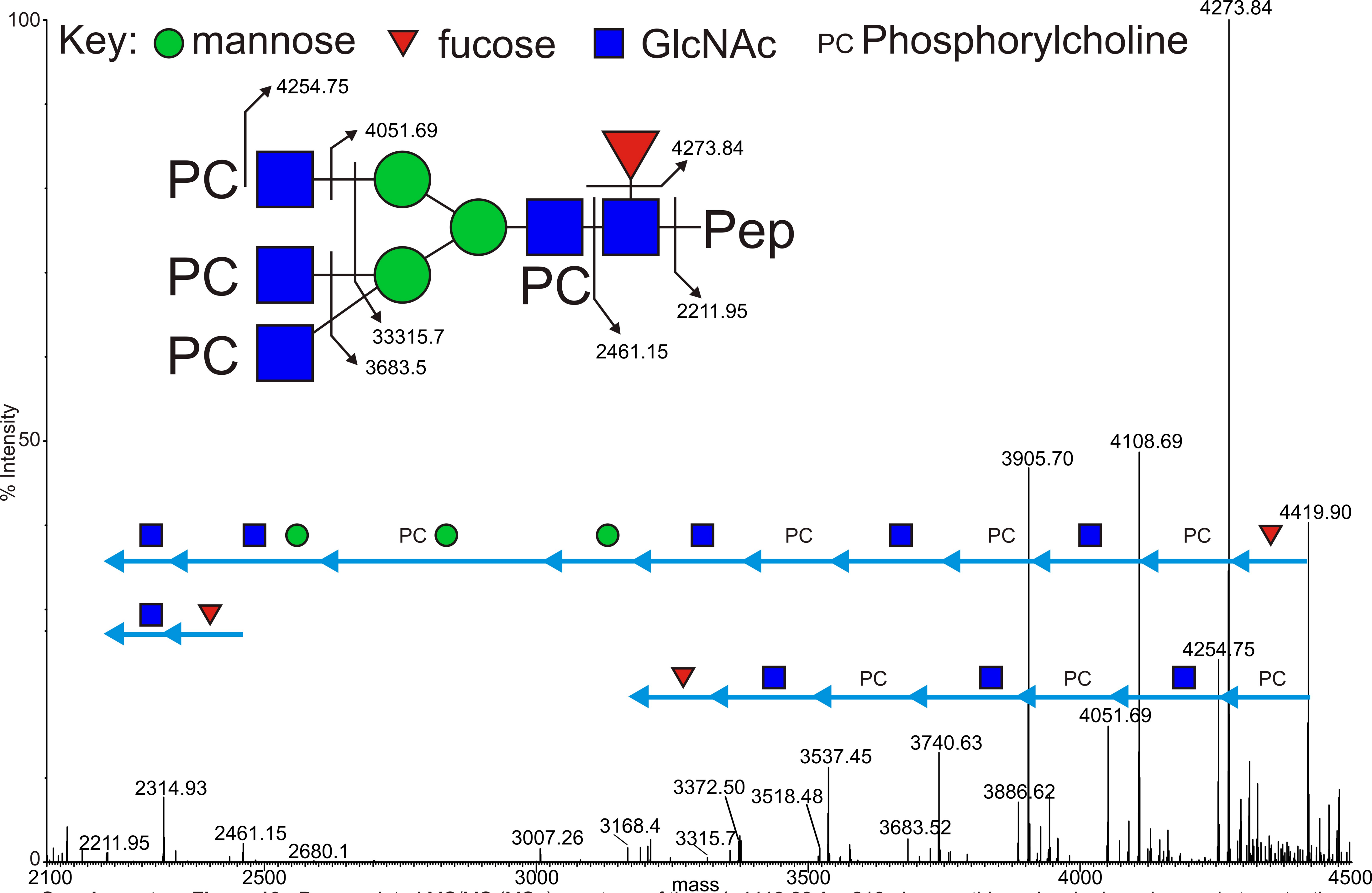

Supplementary Figure 10 - Deconvoluted MS/MS (MSe) spectrum of the *m/z* 4419.83 Asn213 glycopeptide molecular ion, observed at a retention time of 42.6 mins in the reduced, carbamidomethylated chymotrypsin digest of ES-62. Focus is on the high mass region, showing the sequential loss of glycan residues from the intact glycopeptide.

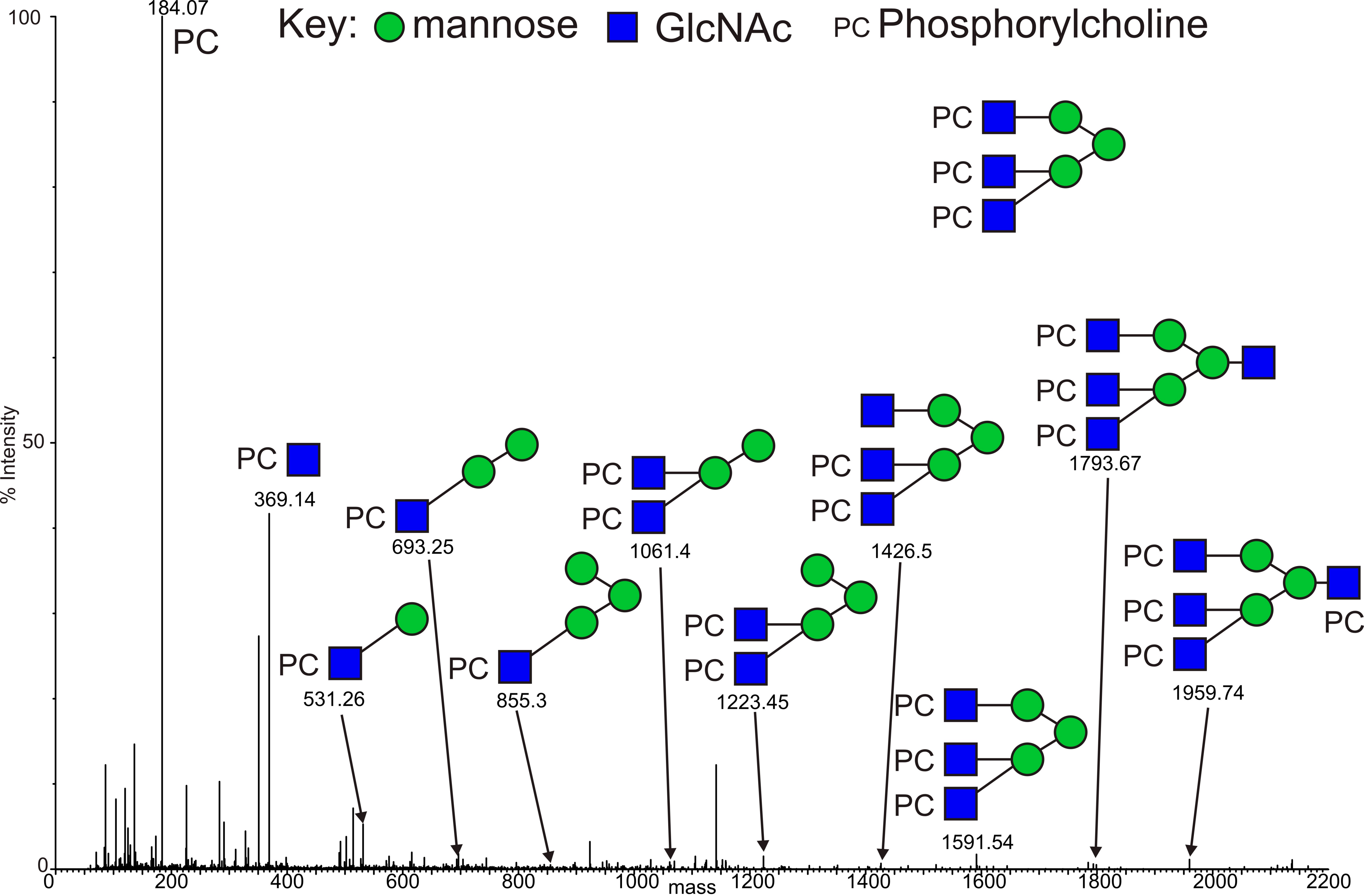

Supplementary Figure 11 - Deconvoluted MS/MS (MSe) spectrum of the m/z 4419.83 Asn213 glycopeptide molecular ion, observed at a retention time of 42.6 mins in the reduced, carbamidomethylated chymotrypsin digest of ES-62. Focus is on the low mass region, showing the fragmented glycan structures.

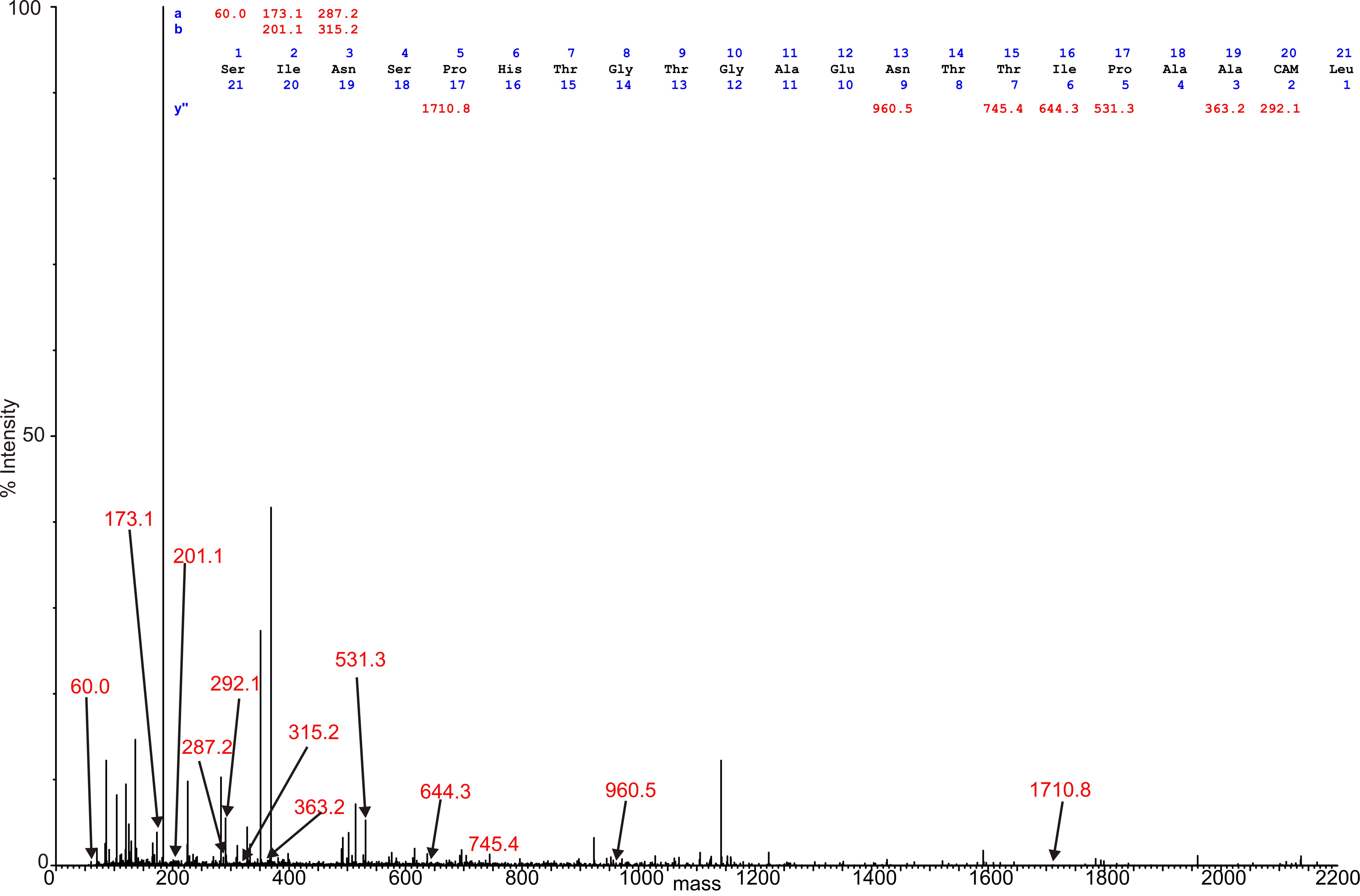

Supplementary Figure 12 - Deconvoluted MS/MS (MSe) spectrum of the m/z 4419.83 Asn213 glycopeptide molecular ion, observed at a retention time of 42.6 mins in the reduced, carbamidomethylated chymotrypsin digest of ES-62. Focus is on the low mass region, showing the peptide fragment ions.
